# Sex Metabolic Differences and Effects on Blood Coagulation Among Rats Exposed to Sodium Dehydroacetate

**DOI:** 10.3389/fphar.2021.727084

**Published:** 2021-09-14

**Authors:** Xin Chen, Fuxing Hao, Meng Zhang, Jinzha Xiao, Weiya Zhao, Zeting Zhao, Yumei Zhang

**Affiliations:** ^1^ Laboratory of Veterinary Pharmacology and Toxicology, College of Veterinary Medicine, Yangzhou University, Yangzhou, China; ^2^ Jiangsu Co-Innovation Center for Prevention and Control of Important Animal Infectious Diseases and Zoonoses, Yangzhou, China; ^3^ Joint International Research Laboratory of Agriculture and Agri-Product Safety, The Ministry of Education of China, Yangzhou University, Yangzhou, China; ^4^ Jiangsu Agri-Animal Husbandry Vocational College, Taizhou, China

**Keywords:** sodium dehydroacetate, metabolism, blood coagulation, cytochrome P450, VKORC1L1, sex difference

## Abstract

Sodium dehydroacetate (Na-DHA), a fungicide used in food, feed, cosmetics, and medicine, has been found to cause coagulation aberration accompanied by the inhibition of vitamin K epoxide reductase (VKOR) in the liver in rats. VKOR complex 1 (VKORC1) and VKORC1 like-1 (VKORC1L1) are two homologous VKOR proteins. Little information is available on the effect of Na-DHA on VKORC1L1 in the liver or VKORC1/VKORC1L1 in extrahepatic tissue and sex differences in Na-DHA metabolism. In the present study, after administration of 200 mg/kg Na-DHA by gavage, significant inhibition of VKORC1 or VKORC1L1 expression in tissues, as well as prolonged prothrombin time (PT) and activated partial thromboplastin time (APTT), were observed. The PT/APTT in the Na-DHA-exposed males were 1.27- to 1.48-fold/1.17- to 1.37-fold, while the corresponding values in the Na-DHA-exposed females were 1.36- to 2.02-fold/1.20- to 1.70-fold. Serum or tissue Na-DHA concentrations were significantly higher in females than in males. The pharmacokinetic parameters (t_1/2_, C_max_, AUC_0∼24 h_, and MRT_0∼24 h_) of Na-DHA in female rats were significantly higher than those in male rats. Furthermore, cytochrome P450 (CYP) activity was investigated using the cocktail probe method. The results revealed that Na-DHA exhibited an inductive effect on CYP1A2, 2D1/2, and 3A1/2 activities by changing the main pharmacokinetic parameters of probe drugs in male rats. However, no significant change in CYP2E1 activity was found. There were sex differences in the metabolism and coagulation in rats exposed to Na-DHA. The lower metabolism and higher blood Na-DHA concentration in females may be the reasons for higher coagulation sensitivity in female rats.

## Introduction

Blood coagulation is driven by vitamin K (VK)-dependent proteases. The formation or function of VK-dependent clotting factors is known to be directly affected by the VK cycle (Stafford, 2005). The reduced form of VK is an essential cofactor for the post-translational carboxylation of several clotting factors such as factors II, VII, IX, and X in mammals. Coumarin derivatives (such as warfarin) are known as VK antagonists and are widely used oral anticoagulants in the prevention and treatment of thromboembolic disorders (Johnson et al., 2016; Zirlik and Bode, 2017). The anticlotting mechanism of warfarin is known to involve the inhibition of VK epoxide reductase (VKOR) activity. VKOR is the rate-limiting enzyme involved in the conversion of VK epoxide to VK in the VK cycle (Wilson et al., 2003; Tie and Stafford, 2016). VKOR activity was performed by two paralogous subunit protein complexes, VKOR complex 1 (VKORC1) (Oldenburg et al., 2006) and VKORC1-like1 (VKORC1L1) (Rost et al., 2004; Lacombe and Ferron, 2018), in vertebrates including humans, rodents, birds, and fish. VKORC1 has been considered as the main protein supporting VKOR activity in the liver of mammals. VKOR activity in extrahepatic tissues, more or less important according to the tissue, may be partially supported by the VKORC1L1 enzyme, especially in the testis, lung, and osteoblasts (Hammed et al., 2013). VKORC1 and VKORC1L1 were associated with distinct dose-response characteristics and binding sites for oral anticoagulants (Czogalla et al., 2018). In *in vitro* and cell culture models, VKORC1L1 is less sensitive to warfarin compared to the sensitivity of VKORC1 (Lacombe and Ferron, 2018). *Vkorc1* mRNA distribution differs from that of *Vkorc1l1* in mice; *Vkorc1* is strongly expressed in the liver and much less in other tissues, whereas *Vkorc1l1* is more expressed in the brain and testis (Hammed et al., 2013; Caspers et al., 2015). There are species differences in VKOR activity, the expression ratio of VKORC1 and VKORC1L1, and inhibition of VKORC1 and VKORC1L1 by warfarin (Nakayama et al., 2020). The mRNA expression ratios of *Vkorc1* versus *Vkorc1l1* in the liver were 8:1 in turkey and 2:3 in chicken. The maximum velocity of VKOR activity in chickens and ostriches was 3- to 7-fold lower than that in rats (Watanabe et al., 2010).

The main structure of sodium dehydroacetate (Na-DHA), 2H-pyran-2-one, is similar to that of warfarin. Na-DHA is widely used as a preservative in food because of its antimicrobial effect. It is commonly used in baked food and pickled vegetables. Furthermore, it is known to well control *Alicyclobacillus acidoterrestris*-related spoilage in the fruit juice/beverage industry (Cai et al., 2015). Na-DHA is also used in the production of products such as feed, cosmetics, and medicines because of its good anti-mildew and antibacterial effects (Izawa et al., 2018). It has been designated as generally recognized as safe (GRAS) by the US FDA and is widely accepted as a safe food additive in many countries. However, its use as a food additive is disallowed in EU countries, because its adverse effects on human health are unknown (Scordino et al., 2018). The acceptable daily intake of the preservative remains to be established.

Hemorrhage in multiple organs and prolongation of blood coagulation time have been reported in Sprague-Dawley (SD) rats after repeated oral administration of Na-DHA (Sakaguchi et al., 2008). In our previous investigation, it was found that Na-DHA exposure induced coagulation aberrations in Wistar rats along with inhibition of liver VKORC1, the sensitivity to which was higher among female rats than among male rats (Zhang et al., 2017; Chen et al., 2019). The higher serum Na-DHA concentration in female rats indicated that there could be some differences in Na-DHA metabolism or elimination between male and female rats exposed to Na-DHA. Whether VKORC1L1 expression in the liver and extrahepatic tissues or VKORC1 expression in extrahepatic tissue in rats is affected by Na-DHA is not clear. In this study, therefore, we investigated the effects of Na-DHA administration on pharmacokinetic parameters; the activity of metabolism-related enzymes (by using the cocktail probe drug method); and changes in the expression of VKORC1 and VKORC1L1 in different tissues such as the liver, lung, testis, and ovary in Wistar rats. We also aimed to better elucidate the sensitivity of male and female rats to Na-DHA upon exposure by evaluating sex differences in metabolism and blood coagulation profiles.

## Materials and Methods

### Chemicals and Animals

Na-DHA (Cat. No. PHR1609, purity >99.0%) was purchased from Sigma-Aldrich (Shanghai, China). Phenacetin (Cat. No. 100095), omeprazole (Cat. No. 100367, purity = 100.0%), chlorzoxazone (Cat. No. 100364, purity = 99.9%), and dapsone (Cat. No. 100114, purity = 99.2%) were obtained from the Institute of Standard Substances, Chinese Academy of Sciences (Beijing, China). Wistar rats, from the Comparative Medicine Center of Yangzhou University, were housed under the following controlled conditions: temperature of 22°C, relative humidity of 40–60%, and a 12 h light-dark cycle. All experimental protocols were reviewed by the Committee for the Ethics of Animal Experiments of Yangzhou University (Yangzhou, China) and were performed per the Regulations for the Administration of Affairs Concerning Experimental Animals, China, and the EU Directive 2010/63/EU for animal experiments.

### Na-DHA Administration

Na-DHA (200 mg/kg) was administered once daily by gavage for 9∼11 days and normal saline was administered to the control group. The rats were anesthetized by intraperitoneal injection of 2% thiopental sodium. Blood, liver, lung, kidney, and testis or ovary samples were collected for determining Na-DHA concentration by using high-performance liquid chromatography (HPLC) and for analyzing VKORC1/VKORC1L1 expression. Six male and six female rats were used at every sampling point.

### Prothrombin Time and Activated Partial Thromboplastin Time Tests

Blood samples were obtained from the posterior orbital venous plexus of the rats and mixed with trisodium citrate immediately at a volume ratio of 9:1. Plasma was collected by centrifugation and used to determine PT and APTT with a PT assay kit or APTT (ellagic acid type) assay kit (Nanjing Jiancheng Biotechnology Co., Ltd. Nanjing, China) by using a blood coagulation analyzer (Coatron M4; TECO, Nrufahm, Germany) according to the manufacturers’ instructions.

### Determination of Na-DHA Concentration by Using HPLC

HPLC analysis for Na-DHA concentration measurement was based on the method described by Zhang et al. (2016). The blood sample was prepared as described previously. Tissue sample preparation was slightly modified as follows. First, 2.5 ± 0.02 g of tissue and 2.0 g of anhydrous magnesium sulfate were mashed in a mortar and transferred to a 20 ml polytene tube. Acetonitrile (7.5 ml) was added and homogenized (Toly Tron PT 2100, Kinematica, Switzerland) for 2 min. The sides of the blade were rinsed with 2.5 ml of acetonitrile, which was then added to the homogenate. The homogenate was mixed with 100 μl of glacial acetic acid, subjected to vibration for 20 min in an ultrasonic cleaner (KS-250D, Kesheng Equipment Co., China), and centrifuged at 6,000 g (Eppendorf Centrifuge 5804R, Germany) for 15 min. The supernatant was transferred to a 25 ml V-neck bottle. The precipitate was re-extracted with 7.5 ml of acetonitrile in a shaking apparatus (Shanghai Hospital Equipment Ltd., Shanghai, China) for 3 min and in an ultrasonic bath for 20 min, followed by which centrifugation was performed at 6,000 g for 20 min. The resulting supernatant was added to the V-neck bottle. The solvents were evaporated at 45°C in a rotary evaporator with the addition of 2.5 ml of n-propanol. The dry residue was dissolved in 1.0 ml of the mobile phase, centrifuged at 15,000 g for 10 min, and passed through a 0.22 μm organic filter for HPLC injection.

The HPLC conditions were as follows: a Waters X bridge C18 column (5 μm, 4.6 × 250 mm) at 30°C, a mixture (35:65, v/v) of methanol and 0.02% ammonium acetate (pH 5, adjusted with phosphoric acid) was used as the mobile phase at 1.0 ml/min; the injection volume was 20 μl and the detection wavelength was 293 nm.

A standard curve for Na-DHA was y = 200.4x + 8.3425, where y is the Na-DHA chromatographic peak area and x is the Na-DHA concentration in mg/L (*R* ^2^ = 0.9999 at 0.2–10.0 mg/L). The recovery of Na-DHA was 81.94–88.21% and 88.20–93.61% from blank tissue samples spiked with 0.2 mg/kg, and the intra- and inter-day variations were lower than 5.0 and 6.0%, respectively. The limit of detection was 0.2 mg/kg, and the limit of quantification was 0.8 mg/kg.

### qRT-PCR Analysis for *Vkorc1*/*Vkorc1l1*

Isolation of mRNA from excised tissues was performed using the MiniBEST Universal RNA Extraction Kit (TaKaRa Bio Dalian Ltd., Dalian, China) according to the manufacturer’s recommendations. The purity and quantity of the RNA were determined by spectrophotometry using NanoDrop ND-1000 (Thermo Scientific, DE). OD_260/280_ and OD_260/230_ were generally ≥2. Reverse transcription of mRNA was performed using the PrimeScript TM RT Master Mix (TaKaRa) in conjunction with oligo-dT primers and hexanucleotides. Real-time PCR was performed using 2 μl of cDNA and SYBR®Premix ExTaq™ II (Tli RNaseH Plus; TaKaRa) using the ABI Prism 7,700 Sequence detection system and the relative quantification method. All assays were performed in duplicate.

Based on the sequence of rat *Vkorc1*, *Vkorc1l1*, and *β-actin* genes in Gene Bank, the following primers were designed: *Vkorc1* F-5ʹGGACGTTGGGCCTCTATCCT3ʹ, R-5ʹCAACATCAGGCCCGCATTGA3ʹ; *Vkorc1l1* F-5ʹCTCCAGATGGGGTCGAGGATT3ʹ, R-5ʹGCTGGCTGTCATGCCAAGTAA3ʹ; and *β-actin* F-5ʹAGATCAAGATCATTGCTCCTCCTG3ʹ, R-5ʹCGCAGCTCAGTAACAGTCCG3ʹ. All primers were synthesized by Sangon Biotech Co. Ltd. (Shanghai, China). The qRT-PCR cycle program was as follows: 1 cycle of 30 s at 95°C, 40 cycles of 3 s at 95°C, 30 s at 60°C, and 5 s at 72°C, with 1 cycle of final extension for 1 min at 72°C.

### VKORC1/VKORC1L1 Western Blot Analysis

Tissue samples were washed three times with pre-cooled phosphate-buffered saline (PBS); 0.1 g of tissue and 3 ml of pre-cooled PBS were fully ground in a glass homogenizer and transferred to a 10 ml centrifuge tube. Centrifugation was performed at 4,000 rpm for 3 min at 4°C. The deposit was washed with 3 ml of pre-cooled PBS and then centrifuged at 4,000 rpm for 10 min at 4°C. The sediments were lysed in 1 ml of lysis buffer (Beyotime Biotechnology, Shanghai, China) for 30 min on ice. After centrifugation at 4,000 rpm for 10 min at 4°C, the supernatant was collected in a 1.5 ml EP tube and then centrifuged at 12,000 rpm for 10 min at 4°C. The protein concentration of the final supernatant was determined and adjusted, and a constant protein concentration (30 μg/lane) was used. The proteins were separated by 12% sodium dodecyl sulfate-polyacrylamide gel electrophoresis and transferred to a nitrocellulose membrane (Amersham Pharmacia Biotech Inc., New York, United States) by using a Bio-Rad Transblot apparatus (Bio-Rad Laboratories Inc., Hercules, CA, United States). After being blocked with 5% skim milk in PBS containing 0.1% Tween-20 for 1 h at room temperature, the membranes were then incubated overnight at 4°C with primary rabbit polyclonal VKORC1 antibody (GTX47837, Gene Tex Co., CA, United States), rabbit polyclonal VKORC1L1 antibody (ab136611, Abcom Shanghai Ltd. Co., Shanghai, China), or mice monoclonal β-actin antibody. Thereafter, the samples were incubated with anti-rabbit IgG secondary antibody conjugated with horseradish peroxidase for 2 h at room temperature. Immunoreactive proteins were visualized using an enhanced chemiluminescence detection system (Bio-Rad Laboratories Inc., Hercules, CA, United States). The grey levels of the bands were assayed using Gel-Pro Analyzer four software (Bio-Rad Laboratories Inc., Hercules, CA, United States).

### Pharmacokinetic Analysis of Na-DHA

The blood samples were collected from the animals’ jugular vein at 0, 0.083, 0.25, 0.5, 1, 2, 3, 5, 7, 9, 11, and 24 h after Na-DHA (200 mg/kg) was singly administered by gavage. The plasma Na-DHA concentration was detected using the HPLC method described above, and the pharmacokinetic parameters were obtained using the WinNonlin software version 5.3.

### Pharmacokinetics Analysis of the Four Probe Drugs in Rats

#### Administration of Probe Drugs and Sampling

Six male and female rats were randomly divided into the Na-DHA group to which 200 mg/kg Na-DHA was administered once daily by gavage for 5 days and the control group, which was administered normal saline. All rats were administered the probe drugs by gavage at 10 ml/kg at 24 h after last administration, with the concentration of dapsone, phenacetin, and chlorzoxazone being 2 mg/ml and that of omeprazole being 4 mg/ml. Blood samples were collected at designated time intervals (0, 0.083, 0.25, 0.5, 1, 2, 3, 5, 7, 9, 11, 24, 33, 48, 60, and 72 h) from the jugular vein; the sample at 0 h was collected directly before the administration of the probe substances. The plasma samples were stored at −20°C until analysis.

#### HPLC Analysis of the Probe Drugs

The concentrations of the four probe substrates for cytochrome P450 (CYP) isoforms were simultaneously analyzed using an Agilent 1200 HPLC system. The HPLC system included the Agilent C18 column (5 μm, 4.6 × 250 mm). Acetonitrile was used as mobile phase A and 0.02 mM phosphate buffer (pH 5.8) was used as phase B. The gradient procedure was as follows: 0∼11.0 min, 30∼45% A; 11.0∼12.0 min, 45∼30% A; 12.0∼13.0 min, 30% A; temperature of the column box of 30°C; detection wavelength 0∼7.2 min, 293 nm; 7.2∼8.5 min, 245 nm; 8.5∼10.0 min, 302 nm; 10.0∼13.0 min, 280 nm; injection volume of 20 μl; and flow rate of 1.0 ml/min.

The plasma samples for probe drug determination were treated as follows. Acetonitrile (300 μl) was added to 100 μl of the plasma samples and vortexed for 3 min; centrifugation was performed at 14,000 rpm for 15 min, and the clear supernatant was collected. Duplicate extraction of the sublayer was performed by repeat addition of 300 μl of acetonitrile, after which the supernatant was combined and evaporated to dryness by using N_2_. The residue was dissolved in 200 μl of the initial mobile phase through vortexing for 3 min and screening with a 0.22 μm filter for HPLC analysis.

The standard curves of the four probe drugs were determined using the HPLC method and read at 0.15∼5.0 mg/ml for dapsone, phenacetin, and omeprazole and at 0.18∼6.0 mg/ml for chlorzoxazone. The recovery of control plasma spiked with 0.15, 0.5, and 2.5 mg/ml phenacetin, omeprazole, and dapsone, and 0.18, 0.75, and 3.0 mg/ml chlorzoxazone was performed. Furthermore, the intra-day precision and lower limits of quantification (LLQ) of the method used for the four probes were determined. The plasma concentrations of the four probe drugs were determined based on the corresponding curves, and the pharmacokinetic parameters of the drugs were analyzed using WinNonlin5.3 software.

### Statistical Analysis

All data were statistically analyzed by GraphPad Prism8 software and expressed as mean ± SM values. One-way analysis of variance (ANOVA) followed by the Turkey post hoc test or Kruskal-Wallis test was used to assess the significance of the differences; the Pearson method was used for correlation analysis. *p* < 0.05 was considered statistically significant.

## Results

### Difference in PT and APTT Prolongation of Male and Female Rats Treated With Na-DHA

**FIGURE 1:**
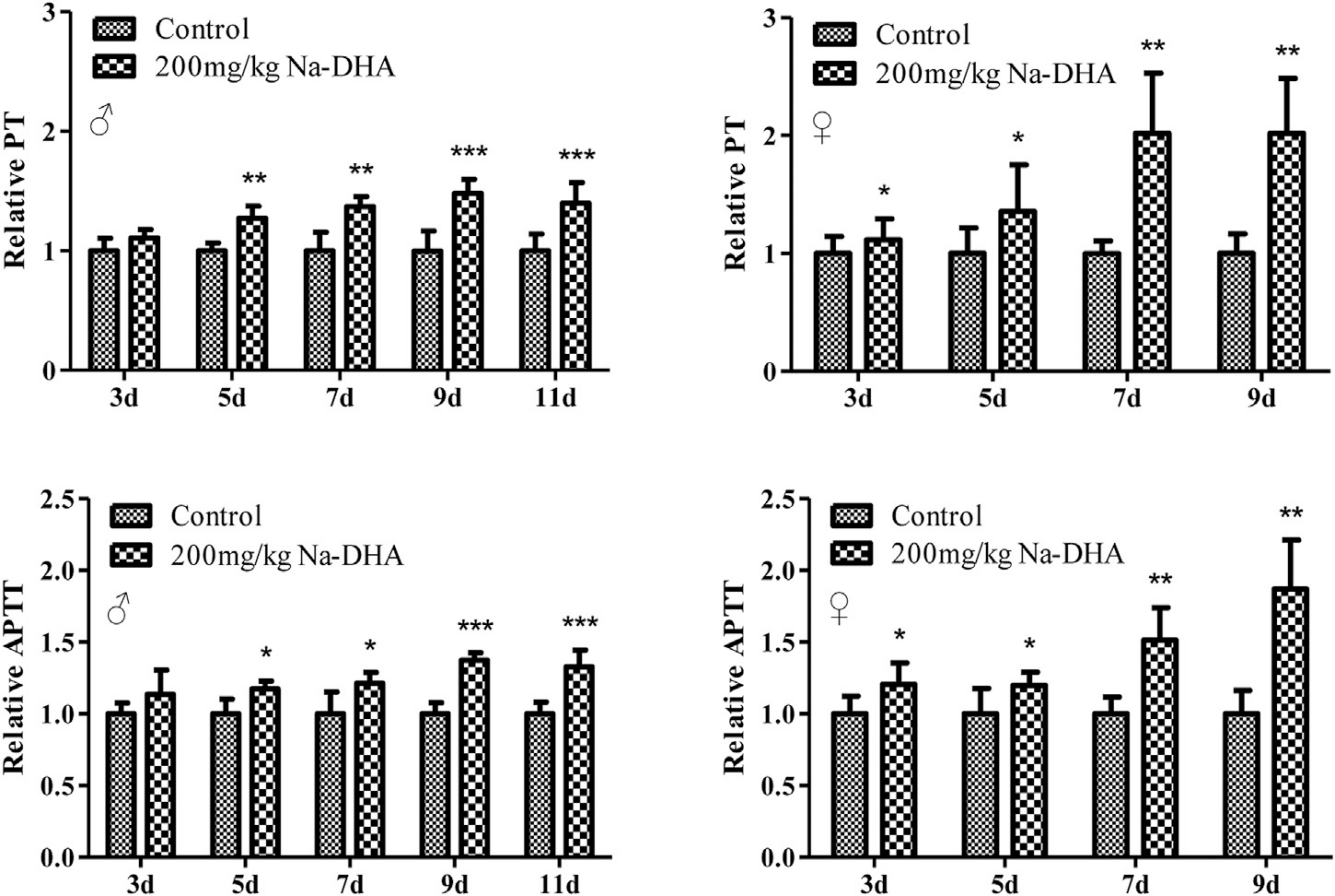
The relative PT and APTT in rats treated with Na-DHA. Wistar rats were administered Na‐DHA at a dose of 200 mg/kg by gavage. PT or APTT values of six males or females in each group were measured by an automated blood coagulation analyzer, and the relative values were compared to those in the controlplasma. *p < 0.05, **p < 0.01, ***p < 0.001, significantly different from controls.

The PT and APTT ratios in the rats treated with 200 mg/kg Na-DHA compared to that in the control group are shown in Figure 1. The PT and APTT in the Na-DHA-treated groups were significantly longer since 5 days after administration (*p* < 0.05). The values of PT ratios were 1.27∼1.48 in males and 1.36∼2.02 in females, the APTT ratio in male rats was 1.17∼1.37 and that in female rats was 1.20∼1.70, compared to the corresponding values in the control group during 7–9 days treatment. PT or APTT were obviously longer in females than in males, especially during days 7–9 of Na-DHA treatment.

### Difference in Serum and Tissue Na-DHA Concentrations Between Male and Female Rats

The Na-DHA concentrations in the serum, liver, lung, and testis of the rats treated by 200 mg/kg are shown in Table 1. At any administration time, the Na-DHA concentrations in serum, liver, and lung of female rats were higher than those in male rats. The concentrations in the female liver and lung were significantly higher than the corresponding concentrations in males at all time points (*p* < 0.05). The Na-DHA concentration ratios in female/male rats were 1.64- to 3.21-fold in the liver, 3.09- to 4.00-fold in the lung, and 1.14- to 1.45-fold in serum during days 3–9 of the treatment. The concentration of Na-DHA in serum achieved a steady-state after 1 week.

**TABLE 1:** Concentrations of Na-DHA in serum and some tissues of rats.

Time(d)	♂				♀		
	Liver (mg/kg)	Lung (mg/kg)	Testis (mg/kg)	Serum (mg/L)	Liver (mg/kg)	Lung (mg/kg)	Serum (mg/L)
3	11.69 ± 1.62^dB^	9.25 ± 0.10^eC^	6.75 ± 0.92^cC^	20.73 ± 0.75^cB^	37.57 ± 4.55^aA^	37.00 ± 1.62^cA^	23.64 ± 1.69^bB^
5	12.21 ± 1.34^dE^	10.76 ± 0.10^dD^	7.29 ± 0.37^bE^	26.20 ± 0.52^bC^	36.00 ± 0.89^aB^	38.34 ± 0.21^cA^	37.86 ± 3.35^bA^
7	13.86 ± 1.51^cD^	14.39 ± 0.18^cD^	8.19 ± 0.71^bE^	31.95 ± 0.96^aB^	26.38 ± 3.96^cC^	44.48 ± 3.87^bA^	45.37 ± 4.63^aA^
9	19.21 ± 1.69^bC^	15.24 ± 0.25^bD^	9.20 ± 1.84^bE^	33.73 ± 0.40^aB^	31.46 ± 5.50^bB^	50.95 ± 0.55^aA^	45.56 ± 2.98^aA^
11	28.52 ± 1.52^aB^	25.99 ± 0.33^aB^	17.00 ± 1.46^aC^	34.32 ± 2.01^aA^			

Note: Na-DHA concentrations in serum are expressed in terms of mg/L and those in the tissues are expressed in terms of mg/kg; data are expressed as the averages of triplet repeat experiments with three rats for each time point. Lowercase letters indicate the same column comparison, and uppercase letters indicate the same row comparison. Same letters indicate that the difference is not significant (*p* > 0.05), and different letters indicate significant differences (*p* < 0.05). The concentration in the ovary was not detected because of the low sample weight.

### Differences in the mRNA Expression Levels of *Vkorc1*/*Vkorc1l1* in Male and Female Rats

**FIGURE 2:**
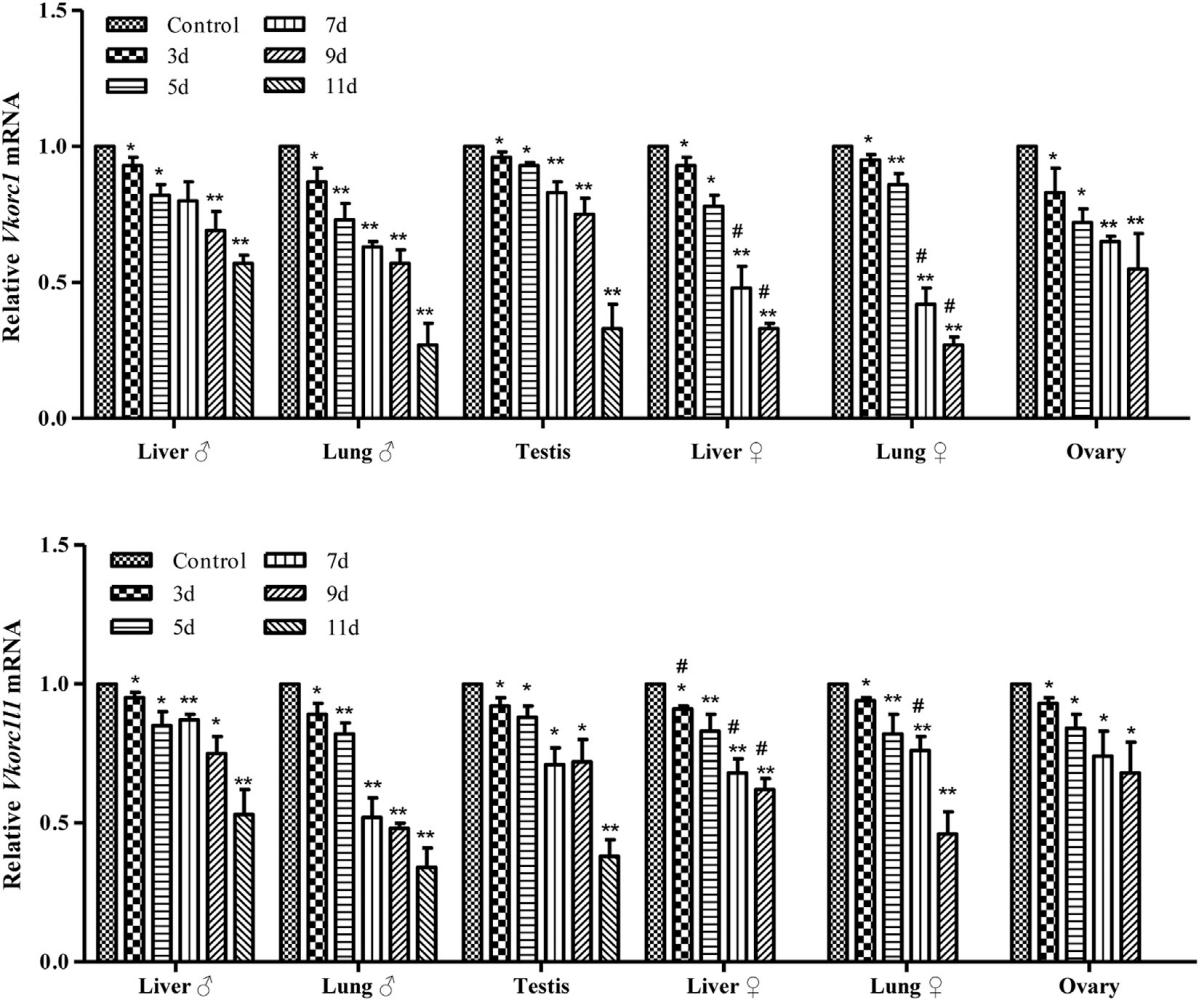
The mRNA levels of Vkorc1/Vkorc1l1 genes in rats treated with Na-DHA. The relative quantification was used in qRT-PCR analysis. The Vkorc1/Vkorc1l1 gene expression in rats treated with 200 mg/kg Na-DHA was normalized with the control in three parallel experiments with triplet replications. *p < 0.05, **p < 0.01, compared with the normal control; #*p* < 0.05, the same tissue in the female and male rats compared at the same time.

The results of qRT-PCR detection of *Vkorc1*/*Vkorc1l1* gene expression in the liver, lung, testis, or ovary of rats treated with 200 mg/kg Na-DHA for different administration times are shown in Figure 2. The mRNA levels of the *Vkorc1*/*Vkorc1l1* genes in different tissues of male or female rats were all significantly reduced at different times of Na-DHA administration (*p* < 0.05 or *p* < 0.01) in comparison with the control. Over 3–9 days of administration, the *Vkorc1* levels relative to those in the control were 0.69- to 0.93-fold and 0.57- to 0.87-fold in the liver and lung of males, and 0.75- to 0.96-fold in the testis; 0.33- to 0.93-fold and 0.27-to 0.95-fold in the liver and lung of females, and 0.55- to 0.83-fold in the ovary, respectively. The *Vkorc1l1* levels were 0.75- to 0.95-fold and 0.48- to 0.89-fold in the liver and lung of males, and 0.72- to 0.92-fold in the testis, respectively. The corresponding levels in the liver, lung, and ovary of females were 0.62- to 0.91-fold, 0.46- to 0.94-fold, and 0.68- to 0.93-fold, respectively. At the same time point of Na-DHA administration, the *Vkorc1* levels in the liver or lung of female rats were lower than the corresponding levels in male tissues, and the differences were significant at 7 and 9 days of administration (*p* < 0.05). The *Vkorc1l1* mRNA levels in the liver of female were significantly lower than those in the male liver at 7 or 9 days of administration (*p* < 0.05).

### Differences in the Expression of VKORC1/VKORC1L1 in Male and Female Rats

**FIGURE 3:**
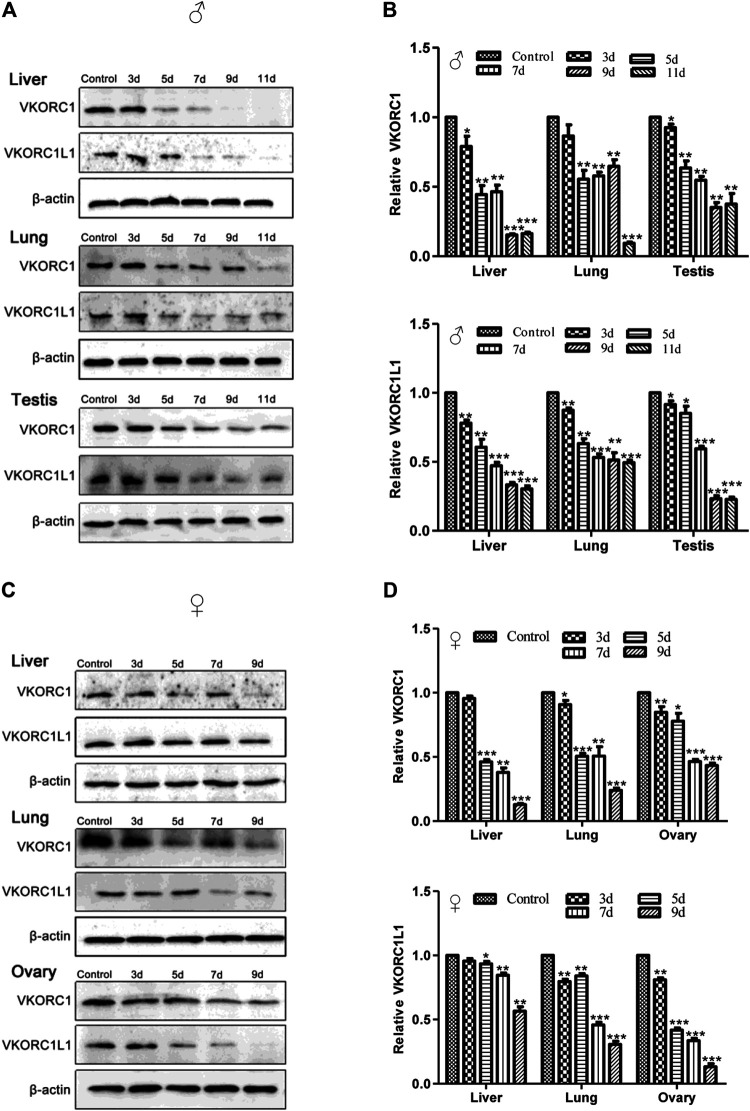
The expression of VKORC1/VKORC1L1 in the tissues of rats treated with Na-DHA. The western blot analysis was performed for the VKORC1/VKORC1L1 expression in tissues of rats treated by 200 mg/kg Na-DHA. A and B for male rats, **(C)** and **(D)** for females. **(A)** and **(C)**, the western blot bands of VKORC1, VKORC1L1 in different tissues of rats; B and D, statistical analysis column of the bands grey values normalized with that in the control over three parallel experiments with triplet repeats. *p < 0.05, **p < 0.01, ***p < 0.001, compared to the normal control group.

The results of western blot analysis for VKORC1/VKORC1L1 expression in the liver, lung, testis or ovary of rats treated by 200 mg/kg Na-DHA for different administration times are shown in Figure 3. The VKORC1 or VKORC1L1 expression in the tissues of male or female rats treated with Na-DHA all showed obvious reductions in comparison with the expression in the control. In male rats, the inhibition of VKORC1 or VKORC1L1 expression in the liver was higher than that in the lung or testis. Furthermore, the relative VKORC1 expression was 0.15- to 0.44-fold, 0.55- to 0.65-fold, and 0.35- to 0.64-fold in the liver, lung, and testis, respectively. The corresponding VKORC1L1 expression during 5–9 days of Na-DHA administration was 0.33- to 0.61-fold, 0.51- to 0.63-fold, and 0.23- to 0.85-fold. Inhibition of VKORC1 or VKORC1L1 in the male lung was lower than that in the liver or testis, and the order of inhibition of VKORC1/VKORC1L1 in male tissues was first in the liver, next in the testis, and third in the lung. As shown in Figures 3C,D, the VKORC1 levels in the liver and lung after 5 days of administration were all lower than half (0.13- to 0.46-fold in the liver, and 0.24- to 0.51-fold in the lung). However, in the liver, VKORC1L1 inhibition (0.56- to 0.93-fold) was obviously lower than that of VKORC1 at the same time points. In the ovary, VKORC1L1 inhibition by Na-DHA was obviously greater than that of VKORC1, with the relative VKORC1L1 and VKORC1 levels being 0.13- to 0.42-fold and 0.43- to 0.91-fold, respectively, over 5–9 days of administration. Similar to the findings for primary organs such as the liver or lung, the VKORC1/VKORC1L1 expression in sex organs such as the testis or ovary were also obviously depressed by Na-DHA.

### Pharmacokinetic Parameters in Male and Female Rats Exposed to Na-DHA

The main pharmacokinetic parameters after a single administration of 200 mg/kg Na-DHA are shown in Table 2. The elimination half life (t_1/2_), C_max_, area under the concentration time curve (AUC_0∼24 h_), and mean residence time (MRT_0∼24 h_) in female rats were significantly higher than those in male rats (*p* < 0.05), while the apparent volume of distribution (Vz) was significantly lower than that in the controls (*p* < 0.05), implying differences in pharmacokinetic parameters between male and female rats exposed to Na-DHA and higher inhibition in female rats than in males in terms of metabolism and elimination.

**TABLE 2:** Pharmacokinetic parameters in male and female rats after a single administration of Na-DHA.

	t_1/2_ (h)	t_max_ (h)	C_max_ (mg/L)	AUC (mg/L*h)	Vz (L/kg)	Cl (L/h/kg)	MRT (h)
♂	18.94 ± 4.27	0.85 ± 0.32	322.19 ± 25.09	7556.82 ± 1537.91	0.51 ± 0.01	0.02 ± 0.01	25.59 ± 6.36
♀	23.22 ± 4.55*	1.03 ± 0.82	406.94 ± 7.92*	13781.96 ± 1405.43*	0.36 ± 0.03*	0.01 ± 0.00	33.55 ± 5.58*

Note: Na-DHA (200 mg/kg) was singly administered to the rats, and blood samples were collected at different time points during 0∼24 h. Pharmacokinetic parameters were analyzed using WinNonlin5.3 software based on the plasma Na-DHA concentration in six male or six female rats, as determined by HPLC.

*p < 0.05, compared to that in males.

### Enzymatic Activity of CYP Isoforms in Male and Female Rats Exposed to Na-DHA

The standard curves of the four probe drugs were y = 116.15x + 2.4972, *R* ^2^ = 0.9999 for dapsone; y = 81.26x + 0.4075, *R* ^2^ = 0.999 for phenacetin; y = 40.117x–0.8312, *R* ^2^ = 0.9993 for omeprazole; and y = 26.836x + 0.1015, *R* ^2^ = 0.9999 for chlorzoxazone. The recovery, intra-day precision, and LLQ of the method are shown in Table 3.

**TABLE 3:** Recovery, intra-day precision, and LLQ of the HPLC method.

	The level of probe spiked in plasma (μg/ml)	Recovery (%)	Intra-day precision (%)	LLQ (μg/ml)
Dapsone	0.16∼2.5	96.2∼98.5	2.0∼5.2	0.04
Phenacetin	0.16∼5.0	90.1∼98.5	2.7∼4.8	0.04
Chlorzoxazone	0.18∼3.0	80.5∼89.8	2.6∼8.5	0.09
Omeprazole	0.16∼2.5	92.1∼100.1	1.5∼4.6	0.08

A cocktail of multiple probe drugs was used to evaluate the effect of Na-DHA on the activity of CYP enzymes in rats. The results of pharmacokinetic analysis of multiple probe drugs in male and female rats are presented in Table 4. The data were expressed as the relative ratio of parameters in the Na-DHA group compared to normal controls because of the sex differences in some CYP isoforms (Agrawal and Shapiro, 1997). In the pharmacokinetics of the phenacetin probe drug, the C_max_ and AUC_0→t_ in male rats were significantly lower than those in females, decreasing by approximately half, respectively, while the Vz in male rats was higher than that in female rats (*p* < 0.05), showing that Na-DHA in multiple dose could significantly speed up the metabolism of phenacetin in males, and Na-DHA had the potential to induce male rat CYP1A2 activity in *in vivo*. The corresponding parameters of omeprazole in males, namely, t_1/2_, t_max_, C_max_, and AUC_0→t_, were significantly lower than those in females (*p* < 0.05), with reductions of about 60% in t_1/2_ and t_max_, 50% in C_max_, and 72% in AUC_0→t_, indicating that Na-DHA induced significant CYP2D1/2 activity in males. With regard to the effect of Na-DHA on rat CYP2E1 isoform, the pharmacokinetic parameters of chlorzoxazone showed no significant difference between male and female rats, except that the t_max_ of chlorzoxazone in males was significantly lower than that in females, implying no effect or a slight effect of Na-DHA on the rat CYP2E1 isoform. The pharmacokinetic analysis of dapsone showed that the t_max_, C_max_, and AUC_0→t_ in males significantly decreased by approximately 67, 54, and 52%, respectively, while the Vz increased about 2.85-fold compared to that in females (*p* < 0.05), indicating that Na-DHA accelerates the metabolism of dapsone in male rats and significantly induces CYP3A1/2 activity in male rats. The pharmacokinetic data of the four probe substances in rats validated sex-related differences in the effects of Na-DHA on the CYP activity of rats in *in vivo*.

**TABLE 4:** Relative pharmacokinetic parameters of probe substances in male and female rats exposed to Na-DHA.

		t_1/2_ (h)	t_max_ (h)	C_max_ (mg/L)	AUC (mg/L*h)	Vz (L/kg)	Cl (L/h/kg)	MRT (h)
Phenacetin CYP1A2	♀	1.40 ± 0.56	2.29 ± 0.69	1.63 ± 0.38	2.45 ± 0.68	0.58 ± 0.12	0.44 ± 0.15	1.53 ± 0.40
	♂	1.47 ± 0.19	1.63 ± 0.48	0.80 ± 0.23*	1.24 ± 0.38*	1.37 ± 0.49*	0.85 ± 0.25	1.45 ± 0.22
Omeprazole CYP2D1/2	♀	1.72 ± 0.43	1.80 ± 0.32	1.80 ± 0.50	3.26 ± 1.23	0.59 ± 0.20	0.48 ± 0.24	1.71 ± 0.31
	♂	0.70 ± 0.08*	0.71 ± 0.19*	0.93 ± 0.19*	0.90 ± 0.36*	0.86 ± 0.25	1.12 ± 0.48	1.67 ± 0.36
Chlorzoxazone CYP2E1	♀	0.92 ± 0.05	1.40 ± 0.48	0.89 ± 0.28	1.17 ± 0.31	0.90 ± 0.32	0.86 ± 0.27	1.01 ± 0.05
	♂	1.62 ± 0.51	0.30 ± 0.00*	0.62 ± 0.22	0.89 ± 0.33	1.90 ± 0.71	1.24 ± 0.42	1.65 ± 0.46
Dapsone CYP3A1/2	♀	1.12 ± 0.54	1.33 ± 0.00	1.73 ± 0.43	2.96 ± 1.14	0.41 ± 0.11	041 ± 0.17	1.47 ± 0.70
	♂	1.32 ± 0.35	0.44 ± 0.18*	0.80 ± 0.20**	0.96 ± 0.39*	1.17 ± 0.34*	0.80 ± 0.33	1.28 ± 0.34

Note: The four probe substances were administered by lavage at 24 h after rats were given 200 mg/kg Na-DHA for five consecutive days, and blood samples were collected at different time points during 0∼24 h. Data in Table 3 show the ratios of pharmacokinetic parameters in Na-DHA-treated animals compared to those in normal controls, based on the pharmacokinetic analysis by Winnonlin5.3 for the plasma probe concentration of six males or six females by HPLC determination.

*p < 0.05, **p < 0.01, compared to females.

## Discussion

### Rationale for this Study

In early study, coagulation abnormality was reported with the inhibition of VKOR activity by Na-DHA (Sakaguchi et al., 2008). Our further research disclosed that Na-DHA inhibited the expression of VKORC1 in the liver of rats (Chen et al., 2019). But the effect of Na-DHA on VKORC1L1, an other protein described to be involved in VKOR activity in some extrahepatic tissues, is unknown. Importantly, we observed that female rats appeared more sensitive to Na-DHA than males in terms of PT and APTT prolongation, the VK level, or Na-DHA concentration in the serum in our previous investigation (Zhang et al., 2017). However, the mechanisms underlying the gender differences remain to be fully elucidated.

### Gender Sensitivity of Rats to Na-DHA

In the current research, female rats showed gradual weight loss, especially after 5 days of administration of 200 mg/kg Na-DHA, whereas male rats showed a gradual increase in weight in the entire experimental period although their average weight was significantly lower than the control at the end of the test. The relative PT or APTT values in female rats were significantly higher than those in male, with the *p*-values for PT and APTT being 0.033 and 0.046 at 7 days and 0.039 and 0.0116 at 9 days of administration in ANOVA analysis. The Na-DHA concentrations in the serum or tissues of females were also higher than those in males (Table 1). These results were consistent with our previous report (Zhang et al., 2016). Although the inhibition of VKORC1 or VKORC1L1 protein expression in female rats by Na-DHA was also found in male rats, the trend of VKORC1 inhibition in the liver or lung of female rats was larger than that in males (Figure 3). We propose that higher concentrations in the blood or tissues of females than in males at the same dose of Na-DHA may be a primary cause of greater sensitivity to Na-DHA among females. It is well known the dose or therapy of warfarin in human are greatly affected by polymorphism altering CYP and VKORC1 genes (Martignoni et al., 2006; Mandic et al., 2015). We deduce the different Na-DHA concentrations in males and females may be mainly related to its metabolic enzyme, such as CYP. We found that Na-DHA concentrations in the tissues rapidly decreased over the Na-DHA withdrawal time, with about 85% of the Na-DHA eliminated from male and female tissues after a 1 week withdrawal period (data not shown), indicating there was no Na-DHA accumulation in tissues. The higher PT or APTT values in females are certainly caused by greater expression inhibition or enzyme activity inhibition of VKORC1 and VKORC1L1. Then, we assessed the metabolic differences between males and females in response to the chemical in our next set of experiments.

### Gender Differences in the Inhibitory Effects of Na-DHA on Both VKORC1 and VKORC1L1

Studies have suggested the presence of species differences in VKOR activity and warfarin inhibition (Wilson et al., 2003; Oldenburg et al., 2015; Nakayama et al., 2020). VKORC1 and VKORC1L1 activity have been confirmed to be different in humans and animals. VKORC1 predominates VKOR activity in the liver, while VKORC1L1 is primarily involved in VKOR activity in extrahepatic tissues. A part of the VKOR activity in extrahepatic tissues supported by VKORC1L1 enzyme was more or less important according to the tissue, especially in the testis, lung, and osteoblasts. In rats, the relative VKORC1 catalytic efficiency was 30-fold greater than that in VKORC1L1, while the VKORC1 catalytic efficiency in humans was 2-fold lower than that of VKORC1L1 (Oldenburg et al., 2015). VKORC1L1 can be inhibited by VK antagonists, but appears to be 50-fold more resistant to VK antagonists than VKORC1, in assessments performed with heterologous expressions of VKORC1 and VKORC1L1 proteins in *P. pastoris* by using pPICZ-VKORC1 or VKORC1L1 vectors individually transformed into the *P. pastoris* SMD1168 yeast strain (Hammed et al., 2013). Although the relationships of VKOR structure and its function have been extensively studied, the functional aspects of VKORC1L1 have not been reported and little was known about this topic until recently.

The differences between VKORC1 and VKORC1L1 expression distribution were also noted in addition to the differences between VKORC1 and VKORC1L1 activity. Studies have shown an obvious difference in the *Vkorc1* and *Vkorc1l1* mRNA distributions in rat or mouse tissues (Hammed et al., 2013; Sinhadri et al., 2017). *Vkorc1* was strongly expressed in the liver, and much lower in other tissues. In comparison with *Vkorc1l1* mRNA, *Vkorc1* was highly predominant in the liver, slightly more in the lung and kidney, and slightly less in the brain and testis. In our present study, absolute quantitative comparisons of *Vkorc1* and *Vkorc1l1* transcription were not performed. We only focused on the relative changes in VKORC1 and VKORC1L1 expression induced by Na-DHA, and the effect of Na-DHA on the two proteins was normalized relative to that in the control group. We found that the effect of Na-DHA on VKORC1 and VKORC1L1 levels of rats was different. The inhibition of VKORC1 or VKORC1L1 expression induced by Na-DHA were not the same in males and females, as well as in different tissues. With regard to the effect on VKORC1 of different tissues, similar inhibition was observed in males or females, with the maximum inhibition in the liver, followed by the lung and then the sex organs (testis or ovary). However, the VKORC1 inhibition rate in the female lung was higher than that in the male lung whereas the inhibition degree in the liver or sex organs in males or females was similar. On the inhibition of VKORC1L1, the inhibition degree in the same tissue in males or females showed greater differences, being slightly more in the liver and sex organ and slightly less in the lung of males in comparison with the corresponding values in females. In male or female rats, the inhibition of VKORC1 was larger than that of VKORC1L1 in the liver and lung and less than that of VKORC1L1 in the testis or ovary (Figure 3). The effect of Na-DHA on the transcription of *Vkorc1* and *Vkorc1l1* genes was not the same as their protein changes. The transcription inhibition of *Vkorc1* and *Vkorc1l1* (with the relative mRNA levels being all larger than 0.5-fold) in the male liver was much far less than the translational inhibition (the relative protein levels being less than 0.5-fold during administration of Na-DHA for 7∼11 days). The inhibition of Na-DHA on *Vkorc1* and *Vkorc1l1* mRNA level in testis and ovary were all far less than their protein level (Figures 2, 3). Based on the effect of Na-DHA on VKORC1 and VKORC1L1, the inhibition of VKORC1 was slightly more than that of VKORC1L1 in the liver and lung and slightly less than that of VKORC1L1 in the testis and ovary, indicating that inhibition of VKORC1 and VKORC1L1 was involved in the abnormal coagulation of rats induced by Na-DHA. The present study is the first to report Na-DHA-induced inhibition of VKORC1L1 and VKORC1 in extrahepatic tissues of rats.

### Gender Differences in the Metabolism of Na-DHA and Probe Drugs in Rats

In our pharmacokinetic analysis of Na-DHA and the four probe drugs, sex differences were observed in the metabolism of Na-DHA as well as the probe drugs. The parameters t_1/2_, C_max_, AUC_0∼24 h_ and MRT_0∼24 h_ in female rats after Na-DHA single administration were significantly higher than those in males, indicating obvious differences in metabolism and elimination of Na-DHA between males and females. The more rapid metabolism of Na-DHA in males or slower metabolism in females was consistent with the lower blood Na-DHA concentration in male blood and higher Na-DHA concentration in female blood. The sex differences in the metabolism of rats exposed to Na-DHA may be related to the effect of the chemical on metabolism enzymes.

CYP enzymes are one of the most important enzymes in drug metabolism and elimination. Many xenobiotics can either inhibit or induce the CYP enzyme (Omura, 1999; Martignoni et al., 2006; Manikandan and Nagini, 2018). Traditionally, studies on drug-drug interactions have been performed by individually evaluating each CYP isoform through probe cocktail substrates (Pillai et al., 2013). The cocktail probe method usually involves simultaneous administration of multiple substrates to determine the activity of multiple CYP enzymes in a single dose (Breimer and Schellens, 1990). The cocktail probe substances are usually phenacetin for CYP1A2 (Floby et al., 2004), chlorzoxazone for CYP2E1 (Kim et al., 2019), omeprazole for CYP2C19 in humans (CYP2D1/2 in rats) (Yin et al., 2016), and dapsone for CYP3A4 in humans (CYP3A1/2 in rats) (Frye et al., 1997; Martignoni et al., 2006). Considering the sex differences in CYP isoforms (Agrawal and Shapiro, 1997), the pharmacokinetic parameters of probe drugs were expressed as relative values of Na-DHA compared to the values of the normal group animals of the same sex in our study. The results of the cocktail probe metabolism showed that the activity of CYP isoforms may be affected by Na-DHA, with different effects on male and female rats. Na-DHA was found to change the activity of CYP1A2, CYP2D1/2 and CYP3A1/2, and cause no or slight changes in CYP2E1 activity. CYP1A2, CYP2D1/2, and CYP3A1/2 were found to be induced in male rats or inhibited in female rats by Na-DHA. Na-DHA also appeared to obviously inhibit the activity of female CYP1A2, CYP2D1/2 and CYP3A1/2 forms in comparison with the male rats. There may be sex differences in the effect of Na-DHA on the CYP1A2, CYP2D1/2 and CYP3A1/2 isoforms. The induction or inhibition of CYP is usually mediated by drugs or xenobiotics, and the effect of Na-DHA on transcription of CYP and its isoforms will be our next work.

### Correlation Analysis of Na-DHA Concentration With PT/APTT

**FIGURE 4:**
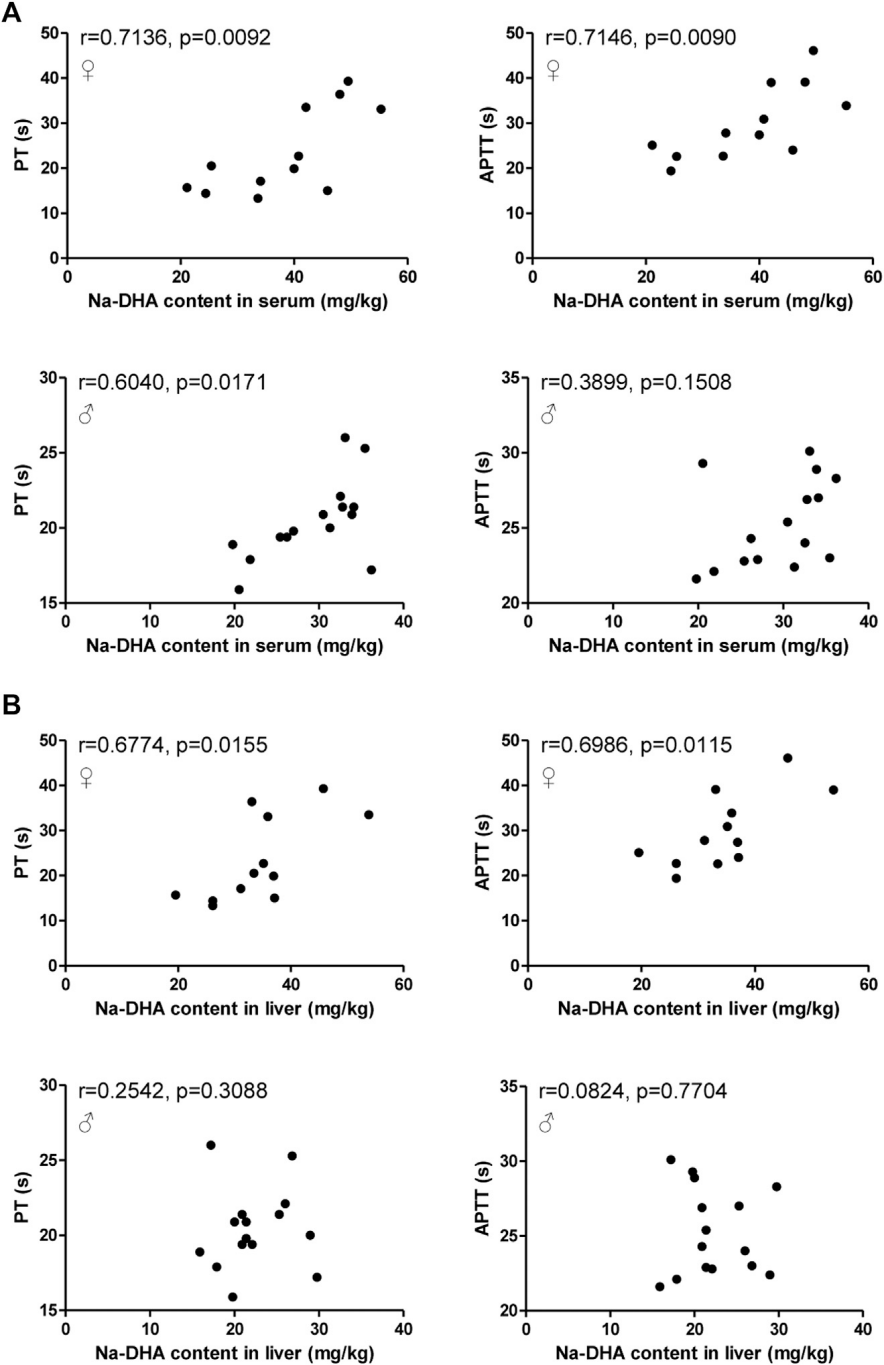
Correlation analysis of Na-DHA content in the blood and liver with PT/APTT. The PT/APTT of five rats administered 200 mg/kg Na-DHA were measured by a coagulation analyzer; the Na-DHA content in the serum and liver of three rats were measured by the HPLC method; **(A)** the correlation analysis of PT/APTT with the serum concentration in male or female rats; **(B)** the correlation analysis of PT/APTT with the Na-DHA concentration in the female liver. Pearson correlation was analyzed. Data were obtained from triplet repeat experiments.

PT and APTT are usually used to evaluate coagulation function. Na-DHA was found significantly lengthen the PT and APTT values in male and females, and appeared to exert an anticoagulation effect. The findings of a correlation analysis of PT/APTT with the Na-DHA levels in blood and liver are presented in Figure 4. The PT or APTT values all showed positive correlations with the Na-DHA concentrations in the serum or liver, and the correlation coefficients of PT or APTT with serum Na-DHA levels in females (r = 0.7136, *p* = 0.0092 for PT; r = 0.7146, *p* = 0.0090 for APTT) were more than those in males (r = 0.6040, *p* = 0.0171 for PT; r = 0.3899, *p* = 0.1508 for APTT), in Figure 4A; the correlation coefficient of Na-DHA in the female liver with the PT or APTT was 0.6774∼0.6986 with significant difference (*p* < 0.05), in Figure 4B, indicating that the Na-DHA concentrations in the liver or in the serum were all better related to coagulation parameter prolongation and appeared to show sex differences.

### Novelty of the Study

This study was set to explain why female rats were more sensitive than male rats to Na-DHA. The findings demonstrate that the effects of Na-DHA on the activity of male CYP enzymes or the higher CYP activities in males than in females resulted in slower metabolism and higher blood concentrations of Na-DHA, longer PT/APTT values, and finally more serious coagulation disorders in females than in male rats.

## Conclusion

We observed sex differences in the metabolism and coagulation function of rats exposed to Na-DHA. Female rats showed more sensitivity to Na-DHA than males may be because of such sex differences in metabolism, effects on CYP enzyme activity, inhibition of VKORC1/VKORC1L1 expression, and prolongation of PT/APTT values.

## Data Availability

The original contributions presented in the study are included in the article/supplementary material, further inquiries can be directed to the corresponding author.
